# Early vs. deferred catheter ablation of ventricular tachycardia in patients of ischaemic substrate: systematic review and meta-analysis of clinical outcomes

**DOI:** 10.1093/ehjopen/oeaf076

**Published:** 2025-06-19

**Authors:** Abhishek Maan, Maaz Waseem, Alex Carter, Kirtivardhan Vashishtha, Tarvinder Dhanjal, Jacob Koruth, E Kevin Heist

**Affiliations:** Department of Cardiac Electrophysiology, 3000 Arlington Avenue, Toledo, OH 43604, USA; Department of Health Policy and Economics, London School of Economics & Political Sciences, Houghton Street, London WC2A 2AE, UK; Department of Biological Sciences, CW405, Edmonton, AB, Canada T6G 2E9; Department of Health Policy and Economics, London School of Economics & Political Sciences, Houghton Street, London WC2A 2AE, UK; Icahn School of Medicine, Mount Sinai Hospital, 1450 Madison Ave, New York, NY, 10029, USA; Division of Biomedical Sciences, Warwick Medical School, Coventry CV4 7 AL, UK; Icahn School of Medicine, Mount Sinai Hospital, 1450 Madison Ave, New York, NY, 10029, USA; Massachusetts General Hospital, Harvard Medical School, 55 Fruit Street, Boston, MA 02114, USA

**Keywords:** Ventricular tachycardia, Catheter ablation

## Abstract

**Aims:**

Ventricular tachycardia (VT) ablation has been shown to reduce the recurrence of VT episodes, but the timing of performing VT ablation (early; at the time of implantable cardioverter defibrillator implantation) or (deferred: after the patient has received ICD shocks) remains controversial. The objective is to conduct a systematic review and meta-analysis of published data from randomized controlled trials (RCTs) in patients with ischaemic cardiomyopathy (ICM) with the aim of comparing outcome of VT ablation stratified by procedural timing.

**Methods and results:**

We conducted a meta-analysis of seven landmark RCTs which included patients with ICM who were either at a high risk of VT or experienced VT/ICD shocks. The primary outcome of VT recurrence was compared according to the timing of performing VT ablation (early vs. deferred). In addition, we also compared the secondary outcome of cardiac mortality. Following a comprehensive search strategy, a total of seven RCTs were included within the final analysis. Based on a pooled analysis, early VT ablation was associated with a significant reduction in the primary outcome [pooled odds ratio (OR) of 0.72, 95% confidence interval (CI): 0.55–0.95, *P* < 0.05] in comparison with a ‘deferred VT ablation’ strategy. The cumulative absolute risk reduction (ARR) for the primary outcome was 0.21, and number needed to treat (NNT) to prevent the outcome of VT recurrence was 4.81. Furthermore, the effect size of early VT ablation compared to a deferred VT ablation approach was more pronounced in reduction of ICD shocks in the subgroup of patients with LVEF > 30% vs. those with LVEF < 30% (pooled OR of 0.65, 95% CI of 0.54–0.79, *P* = 0.01). For the secondary outcomes, we observed that an earlier timing of VT ablation was also associated with both a decrease in cardiac mortality (pooled OR of 0.59, 95% CI of 0.43–0.82) and in the subsequent risk of VT storm (pooled OR of 0.63, 95% CI of 0.51–0.78) when compared with a deferred timing. The cumulative ARR for cardiac mortality was 0.07 and NNT was 15.

**Conclusion:**

The findings from this pooled analysis of seven major RCTs suggest that performing early VT ablation may be beneficial in reducing recurrent VT, ICD shocks, and electrical storm and could also improve cardiac mortality. The benefit of performing early VT ablation was greater in patients with LVEF of >30% amongst this ICM cohort.

**Editorial for this article: Eur Heart J Open 2025; https://doi.org/10.1093/ehjopen/oeaf085**

## Introduction

Ventricular tachycardia (VT) commonly occurs in patients with structural heart disease increasing hospitalization and mortality rates attributable to electrical storm (ES) as well as contributing to progressive heart failure.^1^ Because of increased utilization of healthcare resources, driven by need for inpatient hospitalization, VT continues to pose an important global healthcare burden.^2^ Although implantable cardioverter defibrillators (ICDs) are effective in prevention of sudden cardiac death (SCD), occurrence of ICD shocks has been shown to increase mortality and reduce quality of life.^3^ In order to mitigate the risk of ICD shocks, and to reduce the risk of recurrent VT, catheter ablation of VT has evolved as an effective treatment option.^4^ VT ablation, particularly in comparison with anti-arrhythmic therapy and if performed in a timely manner can improve resource utilization by decreasing VT as well as heart failure related hospitalizations.^5^ Although large, multicenter studies have shown that VT ablation is effective in preventing VT recurrences with improvements in short-term mortality, the data on timing of VT ablation (early vs. deferred) remains limited to smaller studies, with conflicting results.^6^ Furthermore, appropriate ‘patient-selection’ as to which patients would derive the most benefit while counterbalancing the risk of procedural complications also remains an issue which might limit findings of large, randomized trials (RCTs) to a real-world clinical practice.^7,8^ Although the literature in this area continues to evolve with publications of newer RCTs, the optimal timing of VT ablation remains debatable. In this meta-analysis, we sought to perform an updated evaluation of the currently published RCTs which have compared an early vs. deferred VT ablation strategy in patients with ischemic cardiomyopathy. The primary aim of this meta-analysis is to examine the difference in clinical outcomes in these two strategies of VT ablation based on the timing of the ablation procedure.

## Methods

### Search strategy and study selection

We performed our systematic review and meta-analysis in accordance with the guidelines outlined as part of the Preferred Reporting Items for Systematic Reviews and Meta-Analyses (PRISMA) guidelines and common protocol which had the consensus of all co-authors.^9^ For further completion, we have also included the full checklist (*Table 1*). The complete protocol with its accompanying details of our meta-analysis is registered on PROSPERO (protocol ID: 610559) https://www.crd.york.ac.uk/prospero/#myprospero (final approval pending at the time of writing this meta-analysis). The components of PRISMA checklist are also discussed in detail in subsequent sections of this manuscript as well.

**Table 1:** Checklist of items for meta-analysis

Section/topic	Item number	Comments	Page number reported on
Title	1	Identify the report as a systematic review, meta-analysis or both	1
Abstract			
Structured summary	2	Abstract covers the content and serves as structured summary	3
Introduction			
Rationale	3	The rationale of meta-analysis is discussed in the introduction session	4
Objectives	4	Provide an explicit statement of questions being addressed with reference to participants, interventions, comparisons, outcomes and study design (PICOS)	4
Methods			
Protocol and registration	5	Indicate if a review protocol exists, and if and where it can be accessed (e.g. Web-address). If possible, then please provide registration information	5
Eligibility criteria	6	Specify study characteristics (PICOS, length of follow-up), and report characteristics used for eligibility, giving rationale	6
Search strategy	8	Present full electronic search strategy for at least 1 database, including if there were any limits; such that it can be replicated	6
Study selection	9	State the process of selecting studies in the meta-analysis	6
Data collection process	10	Describe the method of data extraction from reports (e.g. piloted forms, independently or in duplicate) and any other processes for obtaining and confirming data from investigators.	6
Data items	11	List and define all variables	7
Risk of bias in individual studies	12	Describe the methods used for assessing the risk of bias in individual studies selected	7
Summary measures	13	State the principal summary measures (odds ratio, risk ratio)	7
Synthesis of results	14	Describe the methods of handling data and combining results of studies (if done, e.g. *I*^2^ testing) for each meta-analysis	8
Risk of bias across studies	15	Discuss methodology used for assessment of risk of bias across the studies selected in meta-analysis	
Additional analyses	16	Pre-specify if any additional analyses were done (sensitivity or subgroup analyses)	8
Results			
Study selection		Provide numbers of studies screened, assessed for eligibility, and included in the review, with reasons for exclusions at each stage, ideally with a flow diagram.	9
Study characteristics		For each study, present characteristics for which data were extracted (e.g. study size, PICOS, follow-up period) and provide the citations.	9
Risk of bias within studies		Present data on risk of bias of each study and, if available, any outcome level assessment	10
Results of individual studies		For all outcomes considered (benefits or harms), present, for each study: (i) simple summary data for each intervention group (ii) effect estimates and confidence intervals, ideally with a forest plot	10, Table
Synthesis of results		Present results of each meta-analysis done, including confidence intervals and measures of consistency	10
Risk of bias across studies		Present results of any assessment of risk of bias across studies (see Item 15)	10
Discussion			
Summary of evidence	17	Summarize the main findings of meta-analysis	11–14
Limitations	18	Discuss limitations of the studies included and meta-analysis	14
Conclusions	19	Provide general interpretation of the study along with future directions	15
Funding	20		NA

For the primary source of studies, we use the following databases:

- PubMed (Medline)
- Embase
- Cochrane library database
- https://clinicaltrials.gov/

In addition to the final publication of the RCTs that we have included in our analysis, we also utilized https://clinicaltrials.gov/ for further details of the enrollment, trial protocol and follow-up. The literature search was conducted independently by the 2 co-authors (AM and MW), and we included RCTs/studies that were published in any language including English as the language of majority of the studies from their initial conception date until December 2024. The following keywords were used for our search strategy:

- Ventricular tachycardia, ablation (title/abstract)
- Ventricular fibrillation (title/abstract)
- Implantable Cardioverter Defibrillator (ICD), ICD shocks (title/abstract)
- Electric storm

In addition, we also supplemented our literature search with cross-references from review articles, consensus and guideline documents. For the studies that we selected for our meta-analysis, we then applied the following *PICOS* criteria:

P: Patient population- for this aspect, we only included patients with ischemic cardiomyopathy who underwent VT ablation as part of an RCT.

I: Intervention: VT ablation was the major intervention of interest in our meta-analysis.

C: Comparison: The major aim of our meta-analysis is the ‘timing’ of VT ablation- early vs. deferred VT ablation. The RCTs included in the meta-analysis had defined an early VT ablation as either (a): performed prophylactically either prior to, concomitantly or within 3 months of an ICD implantation or (b): within 2 months after the initial ICD shock.

In contradistinction, deferred VT ablation was defined as the procedure that was performed at least after 2 months of an episode of monomorphic VT (MMVT).

O: Outcome: The primary outcome of our meta-analysis was the incidence of recurrent episodes of sustained VT (regardless of need for ICD therapies). Secondary outcomes of our meta-analysis were ICD shocks, VT storm and cardiac mortality.

For the further steps of our meta-analysis, we excluded the studies which were not randomized in their study design. In addition, we also used the following exclusion criteria: (a): Review articles, (b): Other meta-analyses on similar topics, (c): Case-series, (d): Pre-clinical investigational studies and (e): conference abstracts.

After our initial search (performed by authors, AM and MW), which was based on title and abstract, we then examined the full-text of the eligible studies to ascertain that these met the aforementioned criteria. In particular, we focused on the comparator groups: ICD+ early VT ablation vs. ICD+ deferred ablation. We adjudicated early VT ablation as the ablation procedure that was performed before the patients experienced episodes of VT/or ES; we also considered preventive VT ablation as early VT ablation and deferred VT ablation was defined as ablation performed after the occurrence of VT/ES to treat the episode(s) of VT. After selecting studies using these inclusion and exclusion criteria, any disagreements were resolved after further discussion between AM and MW and EKH. Further details of our search strategy based on the aforementioned PRISMA checklist are outlined in the flow diagram in subsequent section. An application for research approval was submitted to the ethics department at the London School of Economics (LSE) and considering that our meta-analysis only involved secondary data analysis, an approval was waived.

### Quality assessment

We used the Cochrane collaborative tool for assessment of risk of bias.^10^ In particular, we assessed the risk of bias for certain specific domains such as: Selection of patients in RCTs (as in the choice of allocation concealment vs. random sequence generation or blinded selection of randomly assigned envelopes) and further details of randomization in the RCTs (how the RCTs were carried out; such as physician discretion-based decisions regarding crossovers). Considering the procedural nature and timing of VT ablation, which could be evident to both the operator and the patients; all the RCTs included in our meta-analysis were open-label in design.

Furthermore, given that the final inclusion of studies was restricted exclusively to the RCTs in our meta-analysis; we also assessed limitations specific to trial designs such as lack of follow-up, adjudication of clinical outcomes and cross-over between the early and deferred VT ablation necessitated by clinical presentation. The summary of PRISMA checklist that we used for RCTs is summarized in the *Table 1*. To assess the quality of each reported outcome in the respective RCT included in our meta-analysis, we also utilized the GRADEproGDT software (McMaster University).^11^ We classified every domain as either at a ‘high’ or a ‘low’ risk of bias. If the risk of bias could not be assessed, then it was classified to be ‘unclear’.

### Statistical analysis

Categorical variables are reported as percentages, and proportions and continuous variables are reported as mean and standard deviation. As the initial step, we assessed the number of outcome events in each comparator arm (Early VT ablation vs. Deferred VT ablation). Considering the likely variations in operator experience, procedural aspects (pertaining to both mapping and end-points of ablation); we used the random-effects model for our analyses.^12^

As the primary measure of the treatment effect (as in early vs. delayed), we used pooled odds ratios (OR), using DerSimonian and Laird random effect model^13^ and also calculated 95% confidence intervals. A *P*-value of 0.05 was considered to be statistically significant. An OR of <1 was interpreted as the positive impact of early VT ablation vs. deferred VT ablation on the primary outcome of recurrence of VT. To further compare the treatment-estimate of the two ablation strategies, we also calculated absolute risk reduction (ARR) and number needed to treat (NNT) for both primary and secondary outcomes. NNT was defined as the number of patients who required an earlier VT ablation to prevent 1 event of a given clinical outcome.

The degree of heterogeneity was assessed by using the Q-statistics, Tau^2^, and I^2^ statistic which indicated variability amongst the RCTs included in our meta-analysis. An I^2^ of >50% was adjudicated as the marker of significant heterogeneity. To address heterogeneity, we conducted subgroup analyses stratified by LVEF and performed meta-regression analysis to address variability amongst the clinical outcomes reported in various RCTs. Both these analyses, coupled with the primary objective of our meta-analysis which was aimed at comparing timing of ablation as the intervention helped address significant heterogeneity. We used a funnel plot to assess for publication bias and used Egger’s regression test to adjudicate any asymmetry amongst the RCTs included. All the analyses for our meta-analysis were performed using STATA 14 (StataCorp).

### Sensitivity and subgroup analysis

Additionally, we also performed subgroup analysis to assess the difference in effect size of timing of VT ablation on recurrent VT episodes, in patients with left ventricular ejection fraction of ≥30%. Sensitivity analyses were also performed to determine the effect of an individual RCT from the pooled analysis. To further examine this aspect, we used a ‘leave-one-out’ approach; by excluding one study at a time and compared the overall pooled OR (for all the 7 RCTs) with a leave-one-out pooled OR.

## Results

### Study and clinical characteristics

**Figure 1:**
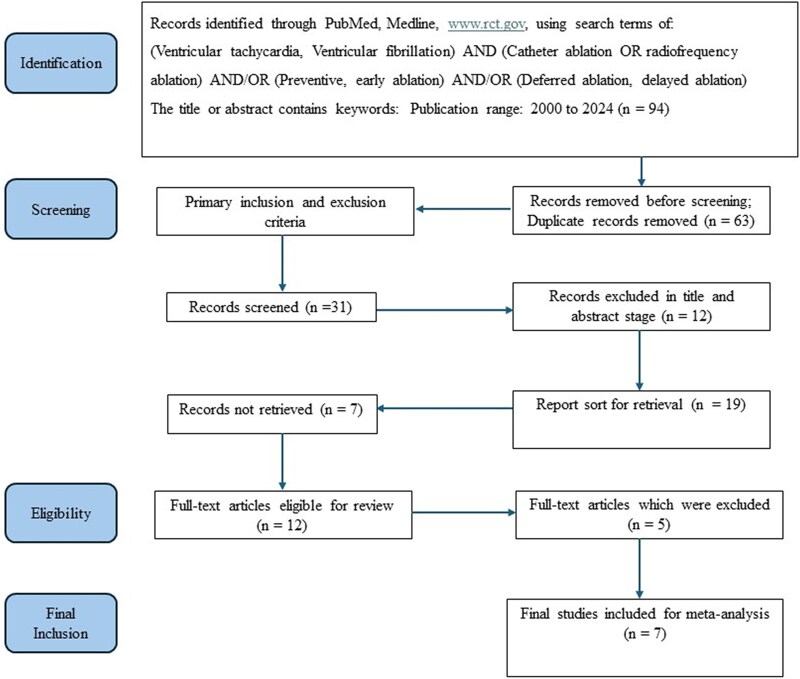
PRISMA flow chart for study selection in our meta-analysis.

A total of 94 studies were identified between 2000 and 2024, based on our search criteria that we previously described. After removing duplicate records (*n* = 63), we then screened the remaining 31 studies for further relevance regarding our meta-analysis. After further detailed full-text screening, we selected 7 RCTs for our final analyses. Our search strategy, study selection and flow-chart leading up to selection of final sample of 7 RCTs is shown in *Figure 1*. It is worth highlighting the geographical and temporal variations in the location of RCTs. One of the earlier RCTs, SMASH-VT was primarily conducted between 2000 and 2006 predominantly in the US,^14^ while the subsequent RCTs such as the VTACH trial in 2010,^15^ and more recent trials such as the SMS,^16^ PARTITA^17^ and BERLIN-VT^18^ trials were conducted in the European Union. From a geographical perspective, the more recent trials such as the PAUSE-SCD and VANISH2 trials had enrolled patients from US and Asia and US and EU region respectively.^19,20^ The study design and baseline characteristics of RCTs included is summarized in *Table 1*. The salient features of the RCTs that were included in the meta-analysis are summarized in the *Table 2*.

**Table 2:** Salient features of randomized controlled trials included in meta-analysis

RCT, year, and sample size	Randomization schema used	Follow-up duration (mean ± SD)	Primary outcome, ARR, and NNT
SMASH-VT 2007 *N* = 128 64 in each arm	Pre-assigned sealed envelopes. No central allocation schema used	22.5 ± 5.5 months	Freedom from any appropriate ICD therapy (either ICD shocks or anti-tachycardia pacing) ARR of 0.20, NNT: 4.9
VTACH 2010 *N* = 107; ablation group = 52, control group = 55	Computer-generated randomly permuted blocks, stratified by centre	22.5 ± 9.0 months	Time to recurrence of VT or VF ARR of 0.19, NNT: 5.3
SMS 2017 *N* = 111; ablation group = 54, control group = 57	Random allocation schema; stratified according to use of amiodarone and beta-blocker therapy	27.6 ± 13.2 months	Time to first recurrence of VT or VF ARR: NA
BERLIN-VT 2020 *N* = 163; preventive ablation = 77, deferred ablation = 86	Computerized central randomization design used. Stratified according to centres and patients	418 ± 277 days in preventive ablation, and 376 ± 290 days in the deferred ablation group	Composite of all-cause death and unplanned hospitalization for either symptomatic VT or HF hospitalization ARR: NA
PARTITA 2022 *N* = 517 (phased screen) Ablation = 23, Standard therapy = 24	Phased randomization. Phase A: Initial observation Phase B: After ICD shock to either VT ablation or standard therapy	Phase A: 2.4 years Phase B: Median follow-up of 24.2 months	Phase A: Appropriate ICD shocks Phase B: Overall mortality, Heart failure hospitalization ARR: 0.37, NNT: 2.7
PAUSE-SCD 2022 *N* = 121 Preventive ablation = 60, Control group of ICD = 61	Randomization using table (block size of 4)	Median of 31.3 months (range of 20.1–40.0 months)	Compose of recurrent VT, cardiac hospitalization, or death ARR of 0.16, RRT: 6.4
VANISH-2 2024 Catheter ablation = 203, Control group = 213	Block randomization, permuted blocks stratified for sotalol or amiodarone	Median of 4.3 years (IQR: 2.5–5.7 years)	Composite of all-cause death, unplanned hospitalization for VT or HF ARR of 0.10, RRT: 10.2

### Clinical outcomes

**Figure 2:**
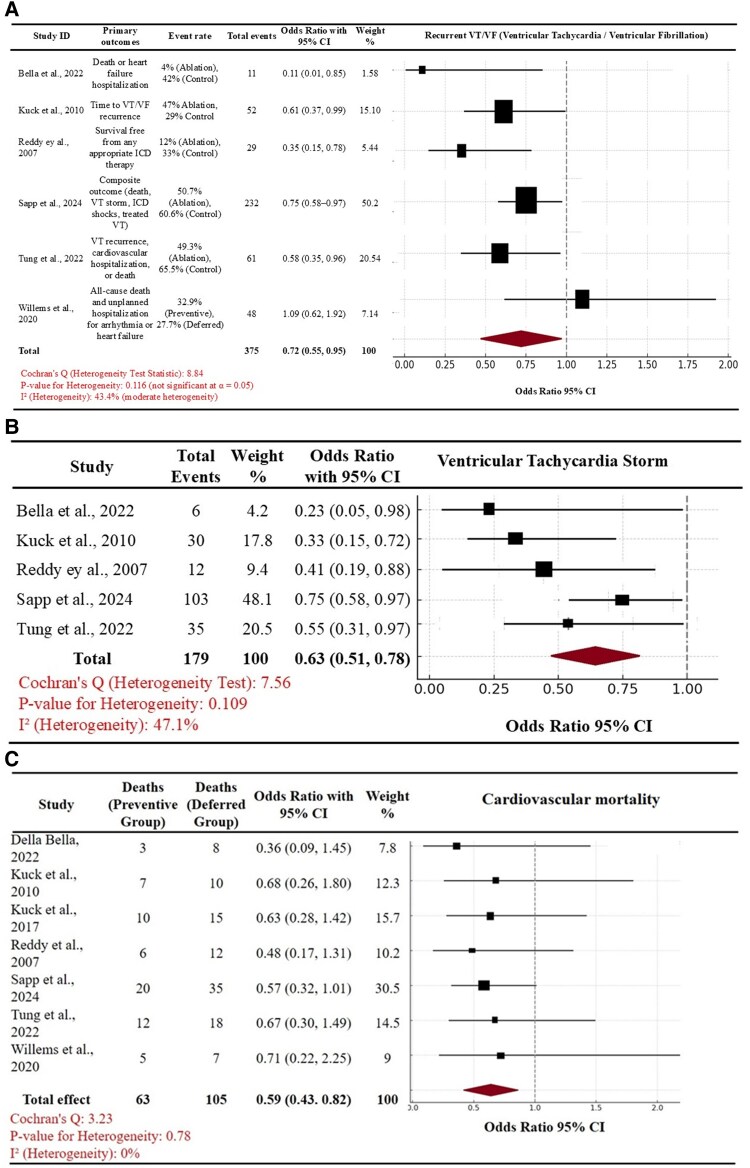
Forest plots comparing pooled odds ratios for recurrent ventricular tachycardia/VF (*A*) and ventricular tachycardia storm (*B*), cardiac mortality (*C*) between preventive vs. deferred ablation in patients with ventricular tachycardia.

The pooled ORs for the primary and secondary outcomes were calculated by the comparison of event rate in the two comparators (early vs. deferred VT ablation). Based on our pooled analysis, early VT ablation was observed to have a beneficial effect in mitigating the risk of recurrent VT/VF in comparison with deferred VT ablation (pooled OR of 0.72, 95% CI 0.55–0.95, *P* < 0.05). We observed that there was at least a moderate degree of heterogeneity (I^2^ of 43.4%, and Cochran’s Q heterogeneity statistic of 8.8) for this outcome. Similar to these findings, we also observed that an earlier timing of VT ablation was associated with reduced risk of VT storm (pooled OR of 0.63, 95% CI of 0.51–0.78) in comparison with a deferred timing of VT ablation. From a comparative perspective, to further assess the association of timing of VT ablation and the primary clinical outcome of recurrent VT, the ARR of 37.3% with early VT ablation was the highest in the PARTITA trial amongst the 7 RCTs. In the most recent VANISH 2 trial, the ARR was 9.8% with an NNT of 10.2. These findings of comparison of early vs. deferred timing of VT ablation on the outcome of recurrent VT, VT storm and cardiac mortality are summarized in *Figure 2A–C*.

**Figure 3:**
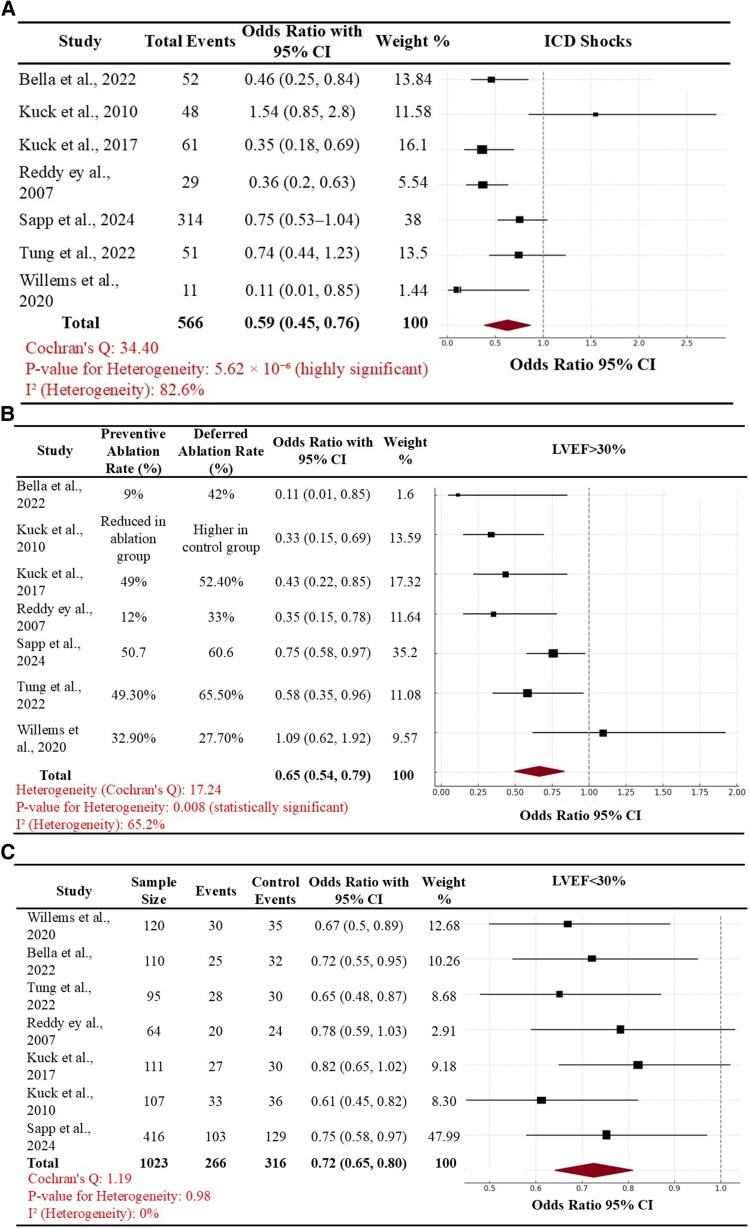
Forest plots comparing pooled odds for the outcome of ICD shocks in the overall population (*A*) and then in the subgroup of patients with left ventricular ejection fraction of >30% (*B*) vs. those with left ventricular ejection fraction of <30% between early vs. deferred ventricular tachycardia ablation (*C*).

Early VT ablation was also effective in mitigating the risk of ICD shocks in comparison to deferred VT ablation (pooled OR of 0.59, 95% CI of 0.45–0.76) and cumulative ARR of ICD shock with an earlier ablation strategy was 0.15 with NNT of 6.6. The beneficial outcome of earlier VT ablation on mitigating the risk of ICD shocks was maintained in patients with LVEF of >30% (pooled OR of 0.65, 95% CI of 0.54–0.79). For this subgroup analysis, we observed a slightly higher, but still a moderate degree of heterogeneity (I^2^ of 65.2% and a Cochran’s Q statistic of 17.2). Considering that we had performed subgroup analysis stratified by LVEF, and timing of ablation, we had observed timing of VT ablation to be the strongest predictor of heterogeneity (*P* = 0.02), which further reinforced our findings of an earlier timing of VT ablation being favorable. Overall, the benefit of an early ablation was more pronounced in patients with LVEF of >30% in comparison to those with LVEF < 30%. The results of these analyses of comparison of early vs. deferred timing of VT ablation on ICD shocks and the stratified sub-analyses according to LVEF are summarized in *Figure 3A–C*.

### Risk of bias assessment

Specifically, we assessed the risk of bias in the studies that were included in our final analysis. Of note, the randomization of patients in the SMASH-VT trial varied from some of more recent trials; as it did not have a central allocation schema. In order to enhance the rate of enrollment, the clinical trial protocol was amended to allow for inclusion of patients with primary prevention ICD who had experienced an ICD shock. VT ablation performed after that ICD shock episode was considered to be deferred VT ablation. It is also worthwhile acknowledging that, there were differences in regard to the clinical threshold in regard to the time of VT ablation. In the more recent, VANISH-2 trial, there were variability in the clinical threshold of sustained VT (as in need for anti-tachycardia pacing or ICD shocks) which necessitated catheter ablation compared to medical therapy.^20^

**Figure 4:**
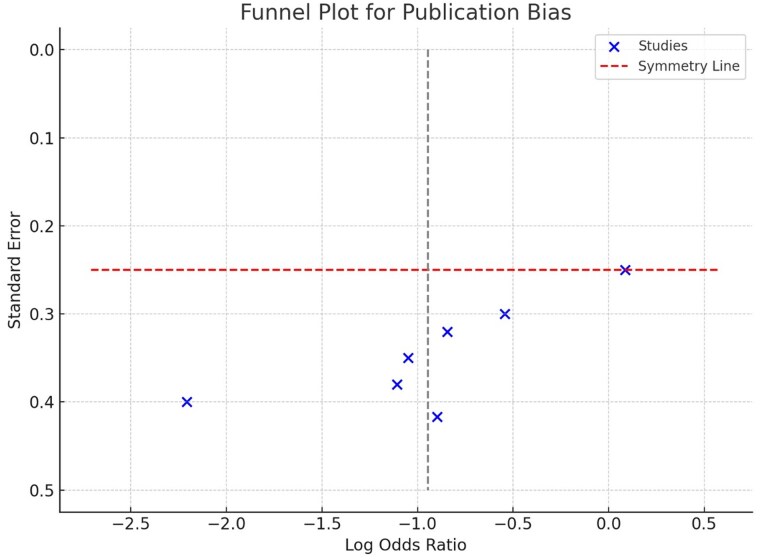
Funnel plot for assessment of publication bias in RCTs comparing preventive vs. deferred ablation for ventricular tachycardia. The plot displays the log odds ratios on the *x*-axis and standard errors on the *y*-axis. Each ‘X’ represents a single RCT, while the dashed line denotes the symmetry line, indicating where studies would align if no bias were present.

There were also at least some differences in regard to the use of anti-arrhythmic drug (AAD) therapy either at the time of enrollment and in conjunction with catheter ablation during the follow-up time period. For example, in the BERLIN-VT trial, 41% of the patients remained on AADs in the early ablation arm in comparison to 27% of patients in the deferred ablation arm. Considering these aspects which include the methods of enrollment coupled with variations in clinical decision being driven in part by physician discretion, it is quite plausible that these factors could contribute to at least a low-risk of bias in the RCT *Table 3*. The summary of risk of bias assessment of the RCTs that we had included in our meta-analysis are summarized in *Table 4* and *Figure 4*.

**Table 3:** Procedural considerations specific to the RCTs included in our meta-analysis

RCT	Procedural strategy	Mapping and ablation strategy	Acute Procedural end-points
SMASH-VT (Reddy *et al.*)	Substrate modification, pace mapping, targeting late potentials, entrainment mapping if VT was haemodynamically stable	Used Non-irrigated RF catheter and Irrigated RF catheter	Noninducibility of VT, and elimination of late and fractionated potentials
VTACH (Kuck *et al.*)	Ablation of clinical VT + scar modification	High-density mapping (either Carto or Ensite velocity) Irrigated ablation	Non-inducibility of VT, loss of capture on pace mapping in scar region
SMS (Kuck *et al.*)	Substrate modification ± non-inducibility of VT	Either high-density or conventional mapping Irrigated ablation	Non-inducibility of VT, or lack of adequate endocardial targets or ineffective lesions
BERLIN-VT (Willems *et al.*)	Ablation of clinical VT + targeting late potentials	High-density mapping (either NavX or Carto), Irrigated ablation	Elimination of late potentials or if radiofrequency ablation time was >1 h
PARTITA (Della Bella *et al.*)	Substrate modification ± activation mapping of inducible VT	High-density mapping (either NavX or Carto), Irrigated ablation	Abolition of late potentials, +non-inducibility of VT after ablation of inducible VT
PAUSE-SCD (Tung *et al.*)	Substrate modification ± activation mapping of inducible VT	High-density mapping (Ensite velocity, Abbott) Irrigated ablation	Abolition of abnormal EGMs within scar and non-inducibility of clinical VT
VANISH-2 (Sapp *et al.*)	Substrate modification ± activation mapping of inducible VT	High-density mapping, Irrigated ablation	Non-inducibility of VT, or abolition of late potentials, and loss of electrical capture with high-output pacing

**Table 4:** Summary of risk of bias assessment in the seven RCTs included in our meta-analysis

Study	Randomization	Blinding	Incomplete outcome data	Other bias	Reporting bias
SMASH-VT	Properly randomized	Blinding of outcome assessors only	Incomplete data due to patient drop-out	Minor bias due to lost follow-ups	Some concerns
VTACH	Randomization with variable block sizes	Blinding of outcome assessors only	Complete data with minimal dropouts	No other significant bias	Low
SMS	Randomized, with stratification	No blinding, open-label study	Complete outcome data available	Potential selection bias	Low
BERLIN- VT	Properly randomized with central allocation	No blinding of participants or personnel	All data included, no missing outcomes reported	No other bias identified	Low
PARTITA	Randomized at multiple centres	No blinding, open-label trial	Complete outcome data reported	No other significant bias	Low
PAUSE-SCD	Randomization confirmed with central allocation	Blinding not specified	Complete outcome data available	No noted bias	Low
VANISH-2	Randomization using block method and stratification for sotalol and amiodarone	Open-label trial, end-point adjudication was blinded	Loss of follow-up in minority of patients (5 in control and 9 in the ablation group)	No noted bias, potential variation in procedural expertise across centres	Low

### Heterogeneity testing/publication bias

Based on I^2^ testing, we did not observe a significant heterogeneity amongst the RCTs. Upon further visual assessment of the Funnel plot, there is some degree of asymmetry based on the deviation as shown in the *Figure 4*. In an ideal scenario, where there is no publication bias; all the studies included in our analysis would be symmetrically distributed along the symmetry line. In these analyses, a few studies are clustered to the left of the symmetry line at lower log odds ratios; which suggests that studies with smaller or negative effects might be under-represented. In our leave-one out sensitivity analysis, we did not observe any significant changes in the pooled treatment effect size upon serially excluding the RCTs which suggests that the overall results of our meta-analysis were not driven by any single study. In our analysis specifically pertaining to the outcome of ICD shocks, we observed at least a moderate degree of heterogeneity, but the most significant factor that influenced heterogeneity was the timing of VT ablation (*P* = 0.02). These findings further reinforce the role of an earlier timing of VT ablation to mitigate the risk of recurrent VT, ICD shocks and VT storm.

## Discussion

This meta-analysis has several key findings which can be summarized as follows:

1. VT ablation, if performed in an early manner (either concomitant, or within 2 months of an ICD implantation; or within 2–3 months of an initial episode of VT) as compared to a deferred manner seems to be significantly effective in mitigating the risk of recurrent VT and ES.
2. An earlier timing of VT ablation also seemed to be more effective in mitigating the risk of ICD shocks as compared to VT ablation that was performed after patients had experienced VT.
3. In comparison to deferred timing of ablation, its earlier timing was also observed to improve cardiac mortality.
4. VT ablation, in particular seems to be effective in the subset with ischemic cardiomyopathy with an LVEF > 30%.

Our meta-analysis includes the more recent trials such as the BERLIN-VT, PARTITA and VANISH-2 trials and supports an earlier timing or preventive approach of VT ablation to mitigate the burden of recurrent VT, ICD shocks. An earlier timing of VT ablation also seems to be favorable for improvement in cardiac mortality as compared to a deferred timing. It is plausible that if the risks of early recurrence of VT and procedural complications after VT ablation could be balanced, then VT ablation might translate to an improvement in outcome of cardiac mortality. Further considering that an early recurrence of VT as compared to delayed recurrence might account as a direct cause of cardiac mortality in these subset of patients.^21^ Albeit not included in our study, as we had restricted our meta-analysis to RCTs; two large single-center studies seem to suggest that VT ablation could translate to improvement in mortality, especially in those patients who might be at high-risk of an early VT recurrence.^21,22^ The benefit of an earlier approach to VT ablation seems more pronounced in patients with an LVEF of >30% and could be potentially explained by a few underlying causes. This subset of patients represents the subgroup which is more hemodynamically compensated and might have a lesser degree of ventricular scar in comparison to those with LVEF < 30%. The findings of better outcomes of VT ablation in those with LVEF > 30% are not surprising. In a study based on 80 patients with ischemic cardiomyopathy, who underwent VT ablation utilizing either a high-density mapping strategy, a higher LVEF was observed to be an independent predictor of success after ablation.^23^

Additionally, a few other studies (albeit some of these have been in the context of specific substrates such as Arrhythmogenic right ventricular cardiomyopathy and non-ischemic cardiomyopathy) have also identified extent of ventricular scar to be an important predictor for VT recurrence. In a study based on 47 of 531 patients with non-ischemic cardiomyopathy, wherein endocardial and epicardial low-voltage regions were carefully annotated, the investigators reported that a greater area of low-voltage characterized upon endocardial unipolar mapping was a significant predictor of recurrence of VT after ablation.^24^ In another study by Avila P, *et al.*, the investigators had assessed the role of non-invasive measurement of scar with pre-procedural cardiac MRI and observed that the presence of (a): larger scar, and (b): heterogeneous distribution of scar were associated with recurrence of VT after ablation.^25^

It is also quite possible that in the patients with LVEF of <30%, an earlier timing of VT ablation might not translate to a reduction in overall cardiac mortality because of a likely progressive pump failure. This finding is also in concert with those from a large prospective epidemiological study by Lee *et al.*, which observed that majority of deaths in patients with advanced cardiomyopathy were due to progressive pump failure and non-cardiac causes and arrhythmogenic causes only accounted for about 15% of overall mortality.^26^ It is also likely that these group of patients might have a larger degree of VT scar as supported by findings of Kojodjojo *et al.*, who based on a large series of VT ablation in 356 patients with ischemic cardiomyopathy had reported scar burden to be an independent predictor for recurrence of VT (adjusted HR of 1.03 for every 3% increase in scar burden, 95% CI of 1.01–1.05, *P* < 0.01).^27^ In the 7 RCTs included in our meta-analysis, although scar burden was considered as a guiding factor to formulate procedural strategy, but it was not evaluated separately as a confounding or predictor of clinical outcomes; particularly recurrent VT after ablation.

It is also worth noting that cardiac mortality was not considered a primary endpoint in majority of the RCTs included in our meta-analysis (SMASH-VT, SMS and VTACH), which could contribute to variability and reporting bias along with its under-estimation as a pooled endpoint.^28^ Although not the primary focus of our meta-analysis, a recent study by Lee *et al.*, based on analysis of patients who underwent VT ablation at Mayo clinic observed that majority of deaths (76%) occurred late after VT ablation and were predominantly due to recurrent VT or pump failure.^29^ Such an observation further lends support to the earlier timing of VT ablation.

From a mechanistic standpoint, it is widely agreed upon that infarcted myocardium serves as an underlying substrate for VT. Considering the differential stages of infarction, edema and scarring; the underlying mechanism of VT in these subsets of patients might encompass all three major mechanisms including triggered activity, automatic and re-entry.^30–32^ Furthermore, there is also evidence of upregulation of potassium voltage-gated channels (KCNE3, KCNE4) at sites within myocardial scarred sites which are at various stages of remodeling after an MI.^32^ Although ICDs are effective at preventing SCD and also at terminating VTs either with anti-tachycardia pacing or with ICD shocks, the substrate for VT remains unmitigated and is often progressive due to remodeling. In routine clinical practice, currently available diagnostic and imaging studies remain limited in their predictive ability to determine the need for an early VT ablation vs. an ablation approach necessitated by failure of AAD therapy and disease progression. Therefore, it might be tempting to favor an earlier VT ablation in a majority of patients, but current evidence to support an upfront preventive approach is limited and there are challenges in regard to appropriate selection of patients who would benefit from such an approach.

Although there is pre-clinical data on complexity and heterogeneity of substrate progression in post-infarct swine models, clinical studies on evolution of border-zone, scar-channels and variation in functional velocity through arrhythmogenic substrate are even more limited.^33^

We also reconcile that there were at least some differences amongst the RCTs included in our meta-analysis. For instance, although the SMS trial did not show a statistically significant difference in the time to event of primary outcome (VT/VF), there was improvement in burden of ICD shocks and VT, VF episodes.^16^ Another additional aspect to consider is the differences in acute endpoints of the procedure (non-inducibility vs. elimination of late and fractionated potentials vs. combined endpoint of both). In the SMASH-VT trial, the procedural end-point was abolition of late and fractionated potentials, but in both the PARTITA and the PAUSE-SCD a combined end point of lack of inducibility and elimination of late and fractionated potentials was adopted. In the latter, remapping was also encouraged to demonstrate electrical quiescence and lack of inducibility. In the VANISH-2 trial; the investigators also aimed for achieving lack of tissue-capture at high-output pacing of >10 mA.

Another point which is worth highlighting is the difference in mapping catheters used (only in the PAUSE-SCD, the investigators used a linear-Duodecapolar catheter) as opposed to the other recent trials (which have used multipolar catheters). Overall, the findings of our meta-analysis are similar to the 2 other previous meta-analyses by Tilz *et al.*^34^ and Kampaktis *et al.*^35^; but there are a few subtle distinctions. Our meta-analysis included recent RCTs such as the PARTITA, BERLIN-VT and the most recent VANISH-2 trial which was published in November 2024. These RCTs had adopted the more contemporary procedural approaches for VT ablation. Considering the inclusion of PAUSE-SCD trial, our meta-analysis also had a more global population as compared to patients from North Americas and EU in rest of the 6 RCTs. We also highlight the nuances of differences in procedural strategies that differed amongst the RCTs.

### Study limitations

We acknowledge several limitations to our meta-analysis. One such limitations pertains to the variability in the definition of an ischemic substrate. The RCTs that we included in our analysis had at least some extent of variability in the definition of an ischemic substrate as well there was heterogeneity regarding adjudication of clinical outcomes (VT, hospitalization and ICD therapies). In regard to the ICD shocks as a follow up outcome, it is quite likely that this might be influenced by differences in device programming and the use of anti-arrhythmic drugs in the follow up period after VT ablation. Both of these factors may be subject to variability based on physician’s discretion in the RCTs that we included. On balance, these factors bear similarities to the practice patterns in a real-world setting where such non-procedural interventions might be necessitated by patient’s presentation. In our meta-analysis, it was not feasible to analyze the variability due to these 2 factors as confounders on the outcome of ICD shocks. Another limitation of our meta-analysis pertains to the limited sample size; particularly in comparison to studies that pertain to atrial arrhythmias which are typically larger in sample size.

In regard to the procedural approach for VT ablation; it is important to note that there could be differences in approaches (substrate modification vs. activation mapping based), use of high-density mapping systems, which could differ amongst the investigators in the RCTs that we had included in our meta-analysis. We also acknowledge that both the mapping and ablation technologies have evolved over the duration of time (especially from 2007 to 2024) over which the RCTs were conducted. Despite these differences in procedural and mapping approaches, it is worth reconciling that majority of the RCTs seemed to have consensus regarding lack of VT inducibility as an acute procedural endpoint.

## Conclusions

Our systematic review and meta-analysis supports that an earlier timing of VT ablation is associated with a significant reduction in the burden of recurrent VT, ICD therapies and electrical VT storm in comparison to a deferred timing of ablation. In carefully selected patients, where the earlier timing of VT ablation can be balanced with procedural complications; such an approach might translate to reduced VT burden and likely benefits in cardiac mortality.

## Data Availability

The data of all the RCTs that we had included in our meta-analysis is available.

## Lead author biography

Abhishek Maan, MD, ScM, MSc, I am an Academic Cardiac Electrophysiologist at University of Toledo; after having trained in Cardiac Electrophysiology at Massachusetts General Hospital in Boston, US. I have also received formal training in quantitative sciences from Brown University and from the London School of Economics & Political Sciences. My clinical focus is on Ventricular tachycardia and catheter ablation and my research efforts are aimed at application of Artificial intelligence and Machine learning to develop algorithms for prediction of sudden cardiac death and VT.
